# Septic Arthritis of the Spinal Facet Joint: Review of 117 Cases

**DOI:** 10.1093/ofid/ofae091

**Published:** 2024-02-14

**Authors:** John J Ross, Kevin L Ard

**Affiliations:** Department of Medicine, Brigham and Women's Hospital, Boston, Massachusetts, USA; Division of Infectious Diseases, Department of Medicine, Massachusetts General Hospital, Harvard Medical School, Boston, Massachusetts, USA

**Keywords:** epidural abscess, facet joint, septic arthritis, *Staphylococcus aureus*, zygapophyseal joint

## Abstract

**Background:**

Septic arthritis of the spinal facet joints is increasingly recognized in the era of magnetic resonance imaging, but its epidemiology, clinical features, management, and prognosis are ill-defined.

**Methods:**

We review 101 previously published cases and report 16 cases occurring at our institutions between 2006 and 2018.

**Results:**

Most patients presented with fever (60%) and back or neck pain (86%). Radiation into the hip, buttock, or limb was present in 34%. The lumbosacral vertebral segments were involved in 78% of cases. Most cases (64%) were due to *Staphylococcus aureus*. Bacteremia was present in 66% and paraspinal muscle abscesses in 54%. While epidural abscesses were present in 56%, neurologic complications were seen in only 9%, likely because most abscesses arose below the conus medullaris. Neurologic complications were more common with cervical or thoracic involvement than lumbosacral (32% vs 2%, *P* < .0001). Extraspinal infection, such as endocarditis, was identified in only 22% of cases. An overall 98% of patients survived, with only 5% having neurologic sequelae.

**Conclusions:**

Septic arthritis of the facet joint is a distinct clinical syndrome typically involving the lumbar spine and is frequently associated with bacteremia, posterior epidural abscesses, and paraspinal pyomyositis. Neurologic outcomes are usually good with medical management alone.

## Teaser

Septic arthritis of the facet joint is a distinct clinical syndrome typically involving the lumbar spine, and frequently associated with bacteremia, posterior epidural abscesses, and paraspinal pyomyositis. Neurologic outcomes are usually good with medical management alone.

The spine contains pairs of true synovial joints between adjacent vertebral bones, known as the *facet joints*. These joints are located in the posterolateral aspect of the spine—except in the high cervical segments, where they are found more anteriorly—and they allow for flexion and extension, rotation, and lateral movement. Although the motion permitted between adjacent vertebral bodies is modest, the sum of these small movements allows for a high degree of spinal mobility as a whole [1].

Prior to the advent of magnetic resonance imaging, septic arthritis of the facet joint was rarely recognized, comprising only 0.1% of cases [2], but it has now increased to 1.1% [3]. The epidemiology, clinical features, and optimal management of facet joint septic arthritis are poorly defined. To address this gap, we review the features of 101 published cases [4–62] and report an additional 16 original cases (Supplementary Table 1).

## METHODS

### Search Strategy and Selection Criteria

We performed a retrospective chart review of adults (age ≥18 years) with facet joint septic arthritis who received care between 2006 and 2018 at 3 teaching hospitals in Boston, Massachusetts: Brigham and Women's Hospital, Brigham and Women's Faulkner Hospital, and Massachusetts General Hospital. The study was approved by the Partners Institutional Review Board (protocols 2017P000348 and 2023P002009). As previously described [3], charts were identified via the Partners Research Patient Data Registry with the following search terms: “septic arthritis,” “infectious arthritis,” “pyogenic arthritis,” “bacterial arthritis,” “gonococcal arthritis,” “tuberculous arthritis,” “mycobacterial arthritis,” and “fungal arthritis.” A total of 7307 records were reviewed, and 1465 cases of culture-positive native joint septic arthritis were confirmed. Sixteen of these fulfilled our case definition of facet joint septic arthritis: a positive culture of synovial fluid from the facet joint or positive culture of blood, cerebrospinal fluid, or paraspinal or epidural abscess fluid, with imaging studies supporting the diagnosis of septic arthritis of the facet joint.

The literature search and data collection and reporting were conducted in accordance with the MOOSE guidelines (Meta-analysis of Observational Studies in Epidemiology) [63]. Previously reported cases of facet joint septic arthritis indexed before 30 November 2022 were identified with the PubMed search engine (National Library of Medicine) via the terms “septic arthritis” and “facet joint.” An overall 91 full-text publications were reviewed and 35 of these were excluded: 11 pediatric cases, 8 reviews not containing original data, 6 cases in which culture results were negative, 3 cases of viral or crystalline facet arthropathy, 1 case not involving the facet joint, and 6 cases in languages other than English or French. An additional 3 additional records not picked by our initial search were identified on subsequent manual reference searches. In all, 59 publications were included in the analysis, describing 101 cases of facet joint septic arthritis.

### Data Extraction and Analysis

Patient records and previously published reports were assessed for eligibility during data extraction through a standardized form. For each record, we collected data with regard to the following: patient sex, age, level and laterality of facet joint involvement, presenting symptoms, causative pathogen and source of positive culture, white blood cell count, erythrocyte sedimentation rate, and C-reactive protein at presentation, as well as maximum temperature, diagnostic imaging study, comorbid medical conditions, complications, treatment, and 30-day survival. Categorical variables were analyzed with chi-square testing.

## RESULTS

The mean age of patients was 60 years, with 79% being ≥50 years of age. Among cases, 57% (64/112) were men and 43% (48/112) were women (*P* = .13 by chi-square). *Staphylococcus aureus* was the most common causative organism, accounting for 64% of cases (Table 1). Overall, gram-positive cocci were responsible for 91%, gram-negative bacilli 5%, and miscellaneous organisms 4%. Bacteremia was present in 66% of cases. Pathogens were isolated from blood alone in 58% of cases, aspirates of synovial fluid or juxta-articular abscesses in 33%, blood and aspirate fluid in 8%, and cerebrospinal fluid in 1%.

**Table 1.** Causative Organisms in 117 Cases of Septic Arthritis of the Facet Joint

Organism	Isolates, No. (%)
Staphylococci	76 (65)
* Staphylococcus aureus*	75 (64)
* *Coagulase-negative staphylococci	1 (1)
Streptococci	27 (23)
* *Group B beta-hemolytic streptococci (*Streptococcus agalactiae*)	8 (7)
* *Group A beta-hemolytic streptococcus (*Streptococcus pyogenes*)	1 (1)
* *Pneumococcus (*Streptococcus pneumoniae*)	4 (3)
* Streptococcus bovis* group	2 (2)
* Streptococcus anginosus* group	3 (3)
* *Other viridans streptococci	7 (6)
* Streptococcus* sp	2 (2)
Enterococci	3 (3)
Gram-negative bacilli	6 (5)
* Escherichia coli*	2 (2)
* Pseudomonas aeruginosa*	1 (1)
* Klebsiella pneumoniae*	1 (1)
* Salmonella* group D	1 (1)
* Yersinia pseudotuberculosis*	1 (1)
Others	5 (4)
* Candida albicans*	1 (1)
* Mycobacterium tuberculosis*	1 (1)
* Pasteurella multocida*	1 (1)
* Parvimonas micra*	1 (1)
* *Polymicrobial	1 (1)

Facet joint involvement was diagnosed by magnetic resonance imaging in 96% of cases and computed tomography in 4%. The lumbosacral vertebral segments were involved in 78% of cases and the cervical and thoracic segments in 11% and 10%, respectively (Table 2). Infection was right-sided in 48% of cases, left-sided in 46%, and bilateral in 7%.

**Table 2.** Septic Arthritis of the Facet Joint: Spinal Level Involved (n = 115)

Facet Joint	Cases, No. (%)
Lumbosacral	90 (78)
* *L4-L5	45 (39)
* *L3-L4	19 (17)
* *L5-S1	15 (13)
* *L2-L3	7 (6)
* *L1-L2	3 (3)
* *L5-L6 (lumbarized S1)	1 (1)
Cervical	13 (11)
Thoracic	12 (10)

Presenting symptoms and signs are summarized in Table 3. Fever ≥38 °C was present in 60% of patients; 86% had back or neck pain. Radiation into the hip, buttock, or limb was present in 34%. Neurologic deficits occurred in 32% of patients with cervical or thoracic involvement but only 2% of patients with lumbar involvement (*P* < .001 by chi-square).

**Table 3.** Presenting Symptoms and Signs (N = 117)

Symptoms and Signs	Cases (%)
Back or neck pain	101 (86)
* *Nonspecific	63 (54)
* *Lateralizing	38 (32)
Fever ≥38.0 °C	70 (60)
Radicular pain ^a^	40 (35)
Neurologic deficits	10 (9)
Altered mental status	4 (3)
Septic shock	3 (3)

^a^Pain radiating into buttock, leg, or arm.

Leukocytosis was present in 57% (40/70) of patients. Erythrocyte sedimentation rate was elevated in 94% (66/70) of patients, with an overall mean value of 78 mm/h. C-reactive protein was elevated in 97% (58/60) of patients, with a mean value of 167 mg/L.

Most common comorbidities are listed in Table 4. Possible predisposing factors included infection at a distant site (22%), diabetes mellitus (19%), and injection drug use (10%).

**Table 4.** Major Comorbid Conditions (N = 117)

Comorbid Condition	Frequency, No. (%)
Infection at a distant site ^a^	26 (22)
Diabetes mellitus	22 (19)
Injection drug use	12 (10)
Spinal osteoarthritis	12 (10)
Open wound	11 (9)
Back strain/injury	8 (7)
Nosocomial ^b^	6 (5)
Cirrhosis	6 (5)
Neoplastic disease	5 (4)
Alcohol use disorder	3 (3)
Immunosuppressive medication	2 (2)
Cat scratch/dog bite	2 (2)
Miscellaneous ^c^	7 (6)

^a^Infections at a distant site included endocarditis (8), septic arthritis (7), periodontal disease (4), cellulitis (3), pyelonephritis (3), and intra-abdominal.

^b^Nosocomial risk factors included facet joint injection (2), epidural catheter, acupuncture, mesotherapy, and steroid injection for herniated disc.

^c^Miscellaneous risk factors included HIV, end-stage renal disease, gout, S1 lumbarization, contact dermatitis, factitious disorder, splenectomy.

The most common contiguous sites of infection (Table 5) were epidural abscesses (56%), paraspinal abscesses (54%), vertebral osteomyelitis (15%), and psoas abscesses (10%). Fifty-one cases of epidural abscess had detailed imaging or operative findings. Forty-five (88%) were posterior (dorsal), complicating 36 lumbar, 7 thoracic, and 2 cervical facet joint infections. Three (6%) were anterior, all as complications of high cervical facet joint infection. Three (6%) epidural abscesses were circumferential (2 lumbar and 1 cervical).

**Table 5.** Contiguous Sites of Infection (N = 117)

Contiguous Infection	Cases, No. (%)
Epidural abscess	65 (56)
Paraspinal abscess	63 (54)
Osteomyelitis	17 (15)
Psoas abscess	12 (10)
Cord compression	8 (7)
Meningitis	4 (3)
Retroperitoneal abscess	2 (2)
Gluteal abscess	1 (1)

### Treatment and Outcomes

Overall 30-day survival was 115 of 117 patients (98%), with both deaths occurring in patients with septic shock and multisystem organ failure.

Of the 107 patients without neurologic deficits, 57 were treated with antibiotics alone; 33 with antibiotics and aspiration of the facet joint, paraspinal abscesses, or both; 7 with antibiotics and surgery; and 10 with antibiotics, aspiration, and surgery. One death occurred in a patient who received antibiotics alone; the remaining patients recovered without sequelae.

Of the 10 patients with neurologic deficits at presentation, 3 made a full neurologic recovery, 5 had partial recovery of motor deficits, 1 had persistent paraplegia due to cord infarction, and 1 died of multisystem organ failure after presenting in septic shock. Of the 3 patients with full neurologic recovery, 2 were treated with decompressive laminectomy and antibiotics, and the other was treated with antibiotics alone. Of the 5 patients with partial neurologic recovery, 4 were treated with decompressive laminectomy and antibiotics, and 1 received antibiotics alone [31, 44, 46, 52, 56]. The patient with persistent paraplegia received antibiotics alone [29], and the patient who died of septic shock received antibiotics and underwent decompressive laminectomy [35].

Data on treatment duration were available for 114 survivors. Seventy-five patients were treated with intravenous antibiotics only, while 39 received a combination of intravenous and oral antibiotics, with no deleterious effect on outcomes. In the 25 patients reported prior to 2000, the mean course of antibiotics was 18 weeks. Among 79 patients reported in 2000 and after, the mean duration of antibiotics was 8 weeks (median, 6 weeks).

## DISCUSSION

Facet joint septic arthritis represents 1% of all septic arthritis cases. It is a distinct clinical syndrome commonly associated with posterior epidural abscesses and paraspinal pyomyositis. This association is likely explained by the intimate anatomic relationship of the facet joint with the posterior epidural space and the paraspinal multifidus muscles. The ligamentum flavum, which forms the posterior boundary of the epidural space, is interwoven with the anterior aspect of the joint capsule, while tendinous fibers of the multifidus muscle reinforce its medial aspect [64].

As the capsule of the facet joint is small, fibrous, and not easily distensible, pyogenic infection would be expected to quickly lead to capsular rupture and spread of pus into the epidural space and paraspinal muscles. Capsular rupture with spread to adjacent structures is common when small volumes of fluid are injected into the facet joint. In one study, when contrast material was injected into a lumbar facet joint to confirm localization during therapeutic corticosteroid injections, total volumes of 2 to 3 mL were associated with epidural spread in 50% of procedures. Contrast leakage into paraspinal muscles was observed in 53% of procedures [65].

Facet joint infection was bilateral in 7% of cases. This likely reflects spread of infection via the retrodural space of Okada. After therapeutic injections, contrast material has been seen to flow from along this potential space from an ipsilateral facet joint to its contralateral counterpart [66].

Bacteremic seeding of a previously damaged facet joint seems to be the likely cause of most episodes of facet joint septic arthritis. The facet joints, with the intervertebral discs, make up the “3-joint complex,” which functions as the major load-bearing unit of the spine [67]. However, our imperfect adaptation to bipedal locomotion makes the lumbar facet joints highly prone to age-related degeneration. Deterioration in the lumbar facets begins at shockingly early ages. More than half of adults aged <30 years have arthritic changes in the lumbar facet joints, such as cartilage wear and osteophyte formation [68]. In fact, these changes precede degeneration of the intervertebral discs [69]. The lumbar facets in which arthrosis is most pronounced at autopsy, L4-L5 and L3-L4 [68], were those most likely to become infected in our study. As the major risk factor for septic arthritis is preexisting joint disease [70], the lumbar facet joints would be expected to be susceptible to infection in the setting of incidental bacteremia.

While epidural abscesses are a frequent complication of facet joint septic arthritis, neurologic outcomes are usually good, likely because most epidural abscesses in this setting occur below the conus medullaris, where the epidural space is more abundant and only nerve roots are present. In our study, neurologic deficits were much more common with infection of the cervical or thoracic facets, as compared with the lumbar facets. This is consistent with the prior literature on the higher risk of neurologic compromise with cervical and thoracic epidural abscesses. In the largest series of spinal epidural abscess, motor deficits were 119% more likely with epidural abscesses proximal to the conus medullaris when compared with more distal epidural abscesses [71].

Directions for future research include the impact of facet joint infection on outcomes in patients with epidural abscess and the optimal treatment of facet joint septic arthritis. The disparate regimens used in this study make it difficult to determine ideal antibiotic duration for facet joint septic arthritis. Many patients treated before the year 2000 received prolonged antibiotics, which seems to be customary in the older literature on septic arthritis [72]. While antibiotic courses of 6 weeks appear adequate, it is unclear whether shorter therapy would also be curative, particularly in the setting of concomitant epidural and paraspinal abscesses.

## CONCLUSIONS

Facet joint septic arthritis represents 1% of all septic arthritis cases. While infection of the facet joint is a less common form of septic arthritis, infectious diseases physicians should be aware of its frequent association with posterior epidural abscesses and paraspinal muscle abscesses, likely because of the intimate anatomic relationship of the facet joint with the posterior epidural space and the paraspinal multifidus muscles. As the major clinical risk factor for facet joint septic arthritis is advancing age and the frequency of facet joint infection by spinal segment correlates with the prevalence of facet joint arthrosis in autopsy studies, it is likely that bacteremic seeding of a degenerated facet joint is the inciting event in most cases. While epidural abscesses are frequent, neurologic outcomes with medical management alone are usually good, due to the posterior location of the abscesses and their occurrence below the conus medullaris in most cases.

## Supplementary Material

ofae091_Supplementary_Data

## Supplementary Data

Supplementary materials are available at *Open Forum Infectious Diseases* online. Consisting of data provided by the authors to benefit the reader, the posted materials are not copyedited and are the sole responsibility of the authors, so questions or comments should be addressed to the corresponding author.

## Notes

***Patient consent.*** The study was approved by the Partners Institutional Review Board (protocol 2023P002009). Informed consent was waived due to the lack of use of identifiable health information and the logistical difficulties in obtaining informed consent in a retrospective chart review study.
