# Supplementary material for: Septic Arthritis of the Spinal Facet Joint: Review of 117 Cases
Source: Open Forum Infect Dis. 2024 Feb 14;11(3):ofae091. doi: 10.1093/ofid/ofae091 (PMC10917203; doi:10.1093/ofid/ofae091)
Supplement: ofae091_Supplementary_Data [file ofae091_supplementary_data.docx]

**Supplementary Table 1. Summary of 16 Original Cases of Facet Joint Septic Arthritis**

| Age/sex | Presenting symptoms | Vertebral level | Organism (culture source) | Other sites of infection | Risk factors/co-morbid conditions | 30-day survival | Treatment |
| --- | --- | --- | --- | --- | --- | --- | --- |
| 66/F | Fever, low abdominal pain | L4-L5 | Methicillin-sensitive *Staphylococcus aureus* (blood) | Posterior epidural abscess | Diabetes mellitus | Yes | Laminectomy with debridement and fusion; 6 weeks IV cefazolin, 6 weeks oral cephalexin |
| 77/M | Fever, hip pain | L4-L5 | *Streptococcus mitis* (blood) | Endocarditis | Gingivitis | Yes | Ceftriaxone IV x 6 weeks |
| 53/F | Buttock pain, low back pain | L5-S1 | Methicillin-sensitive *Staphylococcus aureus* (abscess aspirate) | Paraspinal abscess | Spinal osteoarthritis | Yes | Nafcillin IV x 1 week, oral levofloxacin and rifampin x 7 weeks |
| 69/M | Septic shock | L4-L5 | Methicillin-sensitive *Staphylococcus aureus* (blood) | Endocarditis | Injection drug use | Yes | Nafcillin x 6 weeks, aortic valve replacement |
| 42/M | Low back pain, radiating into hip/leg | L3-L4 | Methicillin-sensitive *Staphylococcus aureus* (blood) | Paraspinal abscess | Injection drug use | Yes | Nafcillin x 6 weeks |
| 82/F | Low back pain | L5-S1 | Methicillin-sensitive *Staphylococcus aureus* (abscess aspirate) | Paraspinal abscess | Cutaneous adenocarcinoma | Yes | Nafcillin x 6 weeks |
| 63/F | Fever, shock | L3-L4 | *Streptococcus agalactiae* (blood, cerebrospinal fluid) | Meningitis, endophthalmitis, posterior epidural abscess, psoas abscesses, vertebral osteomyelitis/diskitis | None | Yes | Penicillin IV x 8 weeks |
| 62/M | Fever, low back pain | L4-L5, L5-S1 | *Streptococcus sanguis* (blood) | Posterior epidural abscess, diskitis | Gingivitis | Yes | Penicillin IV x 6 weeks |
| 76/M | Fever, septic shock | C3-C4 | Vancomycin-resistant *Enterococcus faecium* (blood) | Vertebral osteomyelitis, anterior epidural abscess | Diabetes mellitus, end-stage renal disease, central line-associated bloodstream infection | No | Quinupristin-dalfopristin IV |
| 34/M | Fever, low back pain radiating into leg | L4-L5 | Methicillin-resistant *Staphylococcus aureus* (blood) | Posterior epidural abscess, vertebral osteomyelitis, paraspinal abscess | Injection drug use | Yes | Vancomycin IV x 6 weeks |
| 53/F | Fever, leg pain | C3-C4 | Methicillin-sensitive *Staphylococcus aureus* (blood) | Circumferential epidural abscess, paraspinal abscess | Injection drug use, back injury | Yes | Laminectomy with debridement; nafcillin x 2 weeks (stopped because of rash), vancomycin x 6 weeks |
| 48/M | Low back pain and swelling | L3-L4 | Methicillin-sensitive *Staphylococcus aureus* (abscess) | Psoas, iliacus, gluteal abscesses | Injection drug use, back injury | Yes | Operative drainage, cefazolin IV x 6 weeks |
| 44/M | Low back pain | L3-L4 | Viridans group *Streptococcus* sp. (blood) | Paraspinal abscess | Injection drug use, HIV, cirrhosis | Yes | Ceftriaxone x 2 weeks, doxycycline x 4 weeks |
| 56/F | Fever, low back pain x 3 weeks, thigh cellulitis | L4-L5 | Methicillin-sensitive *Staphylococcus aureus* (bone) | Vertebral osteomyelitis, psoas abscess | None | Yes | Nafcillin IV x 8 weeks |
| 50/M | Fever, low back pain | L4-L5 | *Streptococcus anginosus* (blood, abscess and joint aspirates) | Posterior epidural abscess, paraspinal abscess | Psoriatic arthritis, immunosuppression, back strain | Yes | Laminectomy with debridement, ceftriaxone IV x 4 weeks, amoxicillin po x 4 weeks |
| 36/M | Fever, low back pain | L2-L3 | Methicillin-sensitive *Staphylococcus aureus* (blood, aspirate) | Posterior epidural abscess, meningitis, vertebral osteomyelitis | Spinal surgery with infected hardware | Yes | Hardware removal, cefazolin x 8 weeks, suppressive doxycycline |
